# N-glycosylation is crucial for trafficking and stability of SLC3A2 (CD98)

**DOI:** 10.1038/s41598-022-18779-4

**Published:** 2022-08-26

**Authors:** Lara Console, Mariafrancesca Scalise, Simona Salerno, Raffaella Scanga, Deborah Giudice, Loredana De Bartolo, Annamaria Tonazzi, Cesare Indiveri

**Affiliations:** 1 Department DiBEST (Biologia, Ecologia, Scienze Della Terra) Unit of Biochemistry and Molecular Biotechnology, University of Calabria, Via Bucci 4C, 87036 Arcavacata di Rende, Italy; 2 CNR Institute on Membrane Technology, National Research Council of Italy (CNR-ITM), Via P. Bucci, cubo 17/C, 87036 Rende, Italy; 3 CNR Institute of Biomembranes, Bioenergetics and Molecular Biotechnologies (IBIOM), Via Amendola 122/O, 70126 Bari, Italy

**Keywords:** Biochemistry, Proteins

## Abstract

The type II glycoprotein CD98 (SLC3A2) is a membrane protein with pleiotropic roles in cells, ranging from modulation of inflammatory processes, host–pathogen interactions to association with membrane transporters of the SLC7 family. The recent resolution of CD98 structure in complex with LAT1 showed that four Asn residues, N365, N381, N424, N506, harbour N-glycosylation moieties. Then, the role of N-glycosylation on CD98 trafficking and stability was investigated by combining bioinformatics, site-directed mutagenesis and cell biology approach. Single, double, triple and quadruple mutants of the four Asn exhibited altered electrophoretic mobility, with apparent molecular masses from 95 to 70 kDa. The quadruple mutant displayed a single band of 70 kDa corresponding to the unglycosylated protein. The presence in the membrane and the trafficking of CD98 were evaluated by a biotinylation assay and a brefeldin assay, respectively. Taken together, the results highlighted that the quadruple mutation severely impaired both the stability and the trafficking of CD98 to the plasma membrane. The decreased presence of CD98 at the plasma membrane, correlated with a lower presence of LAT1 (SLC7A5) and its transport activity. This finding opens new perspectives for human therapy. Indeed, the inhibition of CD98 trafficking would act synergistically with LAT1 inhibitors that are under clinical trial for anticancer therapy.

## Introduction

**Figure 1:**
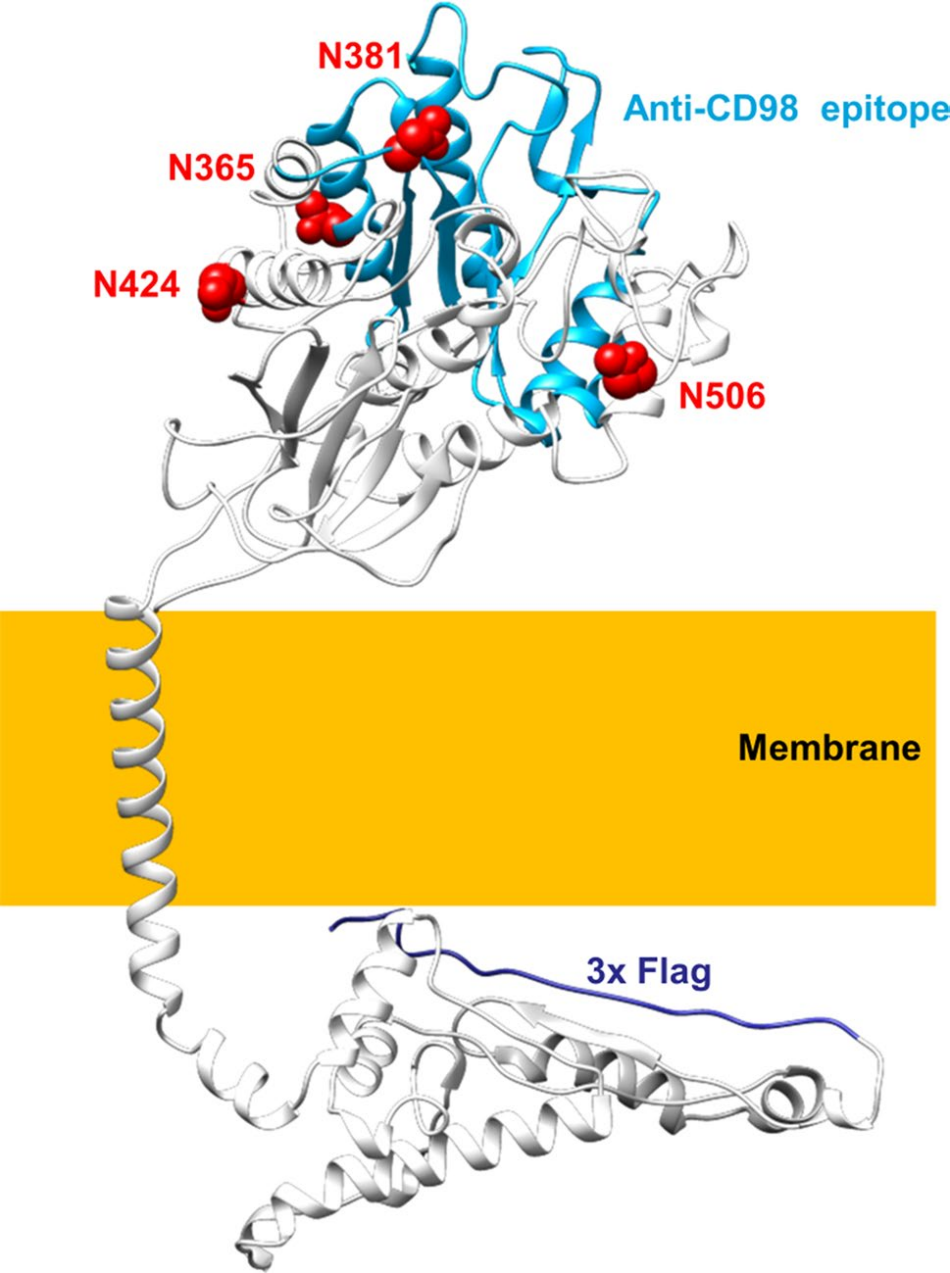
3D model of hCD98. The overall structure of hCD98 viewed parallel to the membrane. The four N-glycosylated residues are represented in a space-filled view and highlighted in red. The epitope recognized by the antibody against CD98 is depicted in light blue while the N-terminal FLAG tag is in dark blue. The membrane is indicated by a yellow box.

The protein CD98, also known as 4F2hc, is a type II glycoprotein formed by an N-terminus intracellular domain, one transmembrane helix, and a large hydrophilic C-terminus portion (Fig. 1). CD98 is ubiquitously expressed in normal tissues and, since the very first studies, it has been found in several tumour cell lines^1^. Even though “CD98” and “4F2hc” are commonly used names, the protein was later included in the third family of the SoLuteCarrier superfamily upon the resolution of the human genome and the evolutionary classification of SLCs. In particular, CD98 is the SLC3A2 member of the family, together with rBAT (SLC3A1). This puzzling naming and classification reflect the sizable number of biological functions identified and characterized over the years for CD98. Indeed, this protein was first identified as a surface antigen typical of activated lymphocytes^2^, and responsible for cell proliferation and growth^3,4^. Then, a link with the pathways of cell adhesion and fusion was proposed, with particular reference to integrin signalling through both the extracellular and the intracellular portions of CD98, modulating mechanical and metabolic signalling^5–9^. Nowadays, the range of CD98-mediated function is even larger and includes: the modulation of inflammation through activation of IL-18 in natural killer cells^10^; the interaction with viral proteins during infections, as in the case of *Herpes simplex* virus^11^; the entry of parasites in cells, such as *Plasmodium vivax* in red blood cells^12^. The most surprising function proposed for CD98 dates back to the early 90’s when the protein was found associated with the uptake of amino acids in Xenopus oocytes^13^. Curiously, this was considered as a secondary function of CD98, ascribable to a covalent association between the so-called CD98 high chain (CD98hc) and a smaller protein, identified as CD98 light chain (CD98lc). Soon after, it was evident that the covalent interaction, between two cysteine residues, was responsible for several metabolic features of cells harbouring CD98^14,15^, and that the identity of the light chain did not correspond to a single protein, but rather to a group of proteins^16,17^. It is now well acknowledged that some of the members of the SLC7 family form functional heterodimers with CD98: (i) system L transporters, LAT1 (SLC7A5) and LAT2 (SLC7A8); (ii) system y^+^L transporters, y^+^LAT1 (SLC7A6) and y^+^LAT1 (SLC7A7); (iii) the system asc transporter, asc-1 (SLC7A10) and (iv) the system xCT (SLC7A11). The biological reason of this interaction is still under debate; indeed, some experimental evidence indicates that CD98 is involved in routing LAT1 to the plasma membrane with no effect on the intrinsic transport function of the protein^18^. Moreover, CD98 seems to be also involved in the stability of LAT2 at the plasma membrane^19^. Then, the recent resolution of LAT1^20,21^ and xCT 3D structures^22^ definitively demonstrated the mutual relationships between CD98 and the SLC7 light chains to form the heterodimer(s) in cell membranes. Some recent reports also indicate that CD98 may modulate the LAT1 transport activity measured in intact cells^21,23^. The terrific interest in CD98 and the cognate proteins began when the link between the CD98-transporter axis and cancer development was described. Indeed, it is well accepted that LAT1 and xCT are key players in cancer metabolism since they are required for essential amino acid supply and redox control, respectively^24–26^. In this respect, knockout of CD98 was shown to reduce the presence of LAT1 at the plasma membrane with alteration of cancer survival^27^. The role of CD98 in cancer was also linked to integrin signalling for cancer survival; indeed, antibodies raised against CD98 were revealed able to impair cancer invasion and metastasis^3,28,29^. Furthermore, CD98 was also shown to interact with PTPRJ, a member of the protein tyrosine phosphatases family, involved in the proteasomal degradation of CD98^30^. Despite the great number of studies dealing with CD98, an important missing piece still exists to complete the picture, that is the trafficking to the definitive location, i.e., plasma membrane. Indeed, understanding this process is fundamental for the complete description of CD98 role in physiological and pathological conditions. The resolution of the 3D structure of the extracellular portion^31^ with the transmembrane domain^20,21^ showed that CD98 has carbohydrate moieties that could be plausibly involved in routing the protein to the plasma membrane. Therefore, we here address this issue by a combined approach of bioinformatics, site-directed mutagenesis and cell biology.

## Results

**Figure 2:**
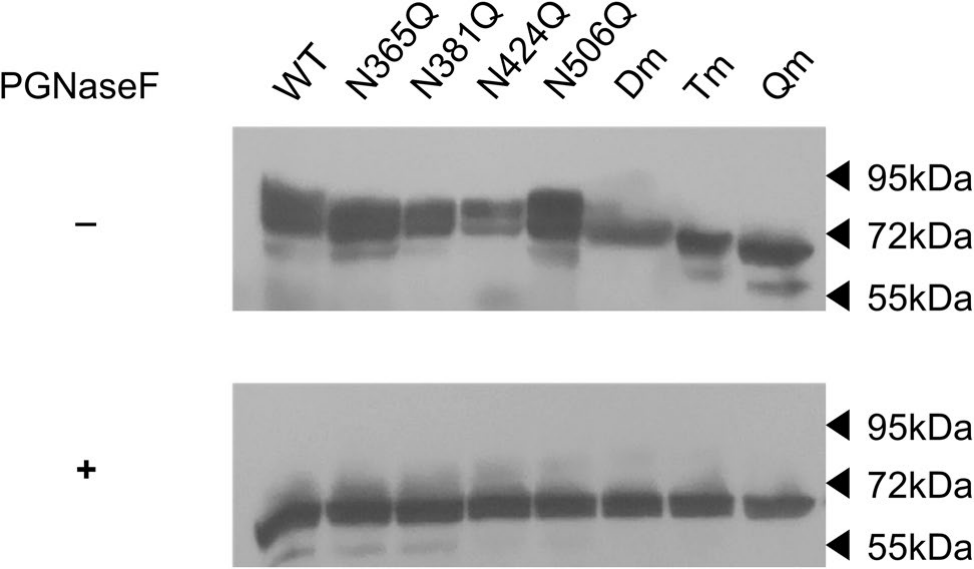
N-glycosidase F effect on CD98. Protein extracts of HEK293 cells transiently transfected with WT-CD98 or mutant-CD98 constructs were subjected to western blot before (upper panel) or after (lower panel) treatment with N-glycosidase F for 3 h at 37 °C. Western blot analysis was carried out using the anti-FLAG antibody^33^. Loading order: WT, N365Q, N381Q, N424Q, N506Q, Dm (double mutant: N381/424Q), Tm (triple mutant: N365/381/424Q), Qm (quadruple mutant: N365/381/424/506Q). The western blot image is representative of three independent experiments.

The presence of N-glycosylated residues in CD98 was deduced based on structural observations and in silico analyses conducted with bioinformatic tools. As shown in Fig. 1, CD98 possesses four Asn residues namely N365, N381, N424, N506 that are predicted as N-glycosylation sites by NetNGlyc-1.0^32^; indeed, the four residues carry glycosyl moieties as demonstrated also by the recently solved LAT1/CD98 complex structure^20,21^. The mentioned Asn residues are located at the surface of the protein, protruding towards the extracellular matrix. The presence of N-glycosylated residues correlates well with the higher apparent molecular mass with respect to the theoretical mass of the sole protein moiety of CD98 (Fig. 2). To investigate the possible role of the N-glycosyl moieties in the trafficking towards the cell membrane, we produced site-directed mutants of CD98 in which each of the N residues, carrying the glycosyl moieties, was substituted with Q. Each mutant was tagged at the N-terminus with FLAG. Moreover, to obtain a glycosyl-free protein, the quadruple mutant (Qm) was constructed by passing through double (Dm: N381/424Q) and triple (Tm: N365/381/424Q) mutants. From the WB analysis, it appears that the loss of a single glycosylated site causes only small changes in the apparent molecular mass of CD98, whereas Dm, Tm and Qm mutants showed a more evident shift of the apparent molecular mass compared to WT (Fig. 2 upper panel). After treatment with PNGase, which specifically removes all the N-linked glycosyl moieties, both WT and mutants showed the same molecular mass of 70 KDa, close to the theoretical mass of the CD98 protein moiety. This finding confirmed that the four identified N residues are the only competent in N-glycosylation of hCD98 (Fig. 2 lower panel), in agreement with the prediction (Fig. 1) and the 3D structure data^20,21^.

**Figure 3:**
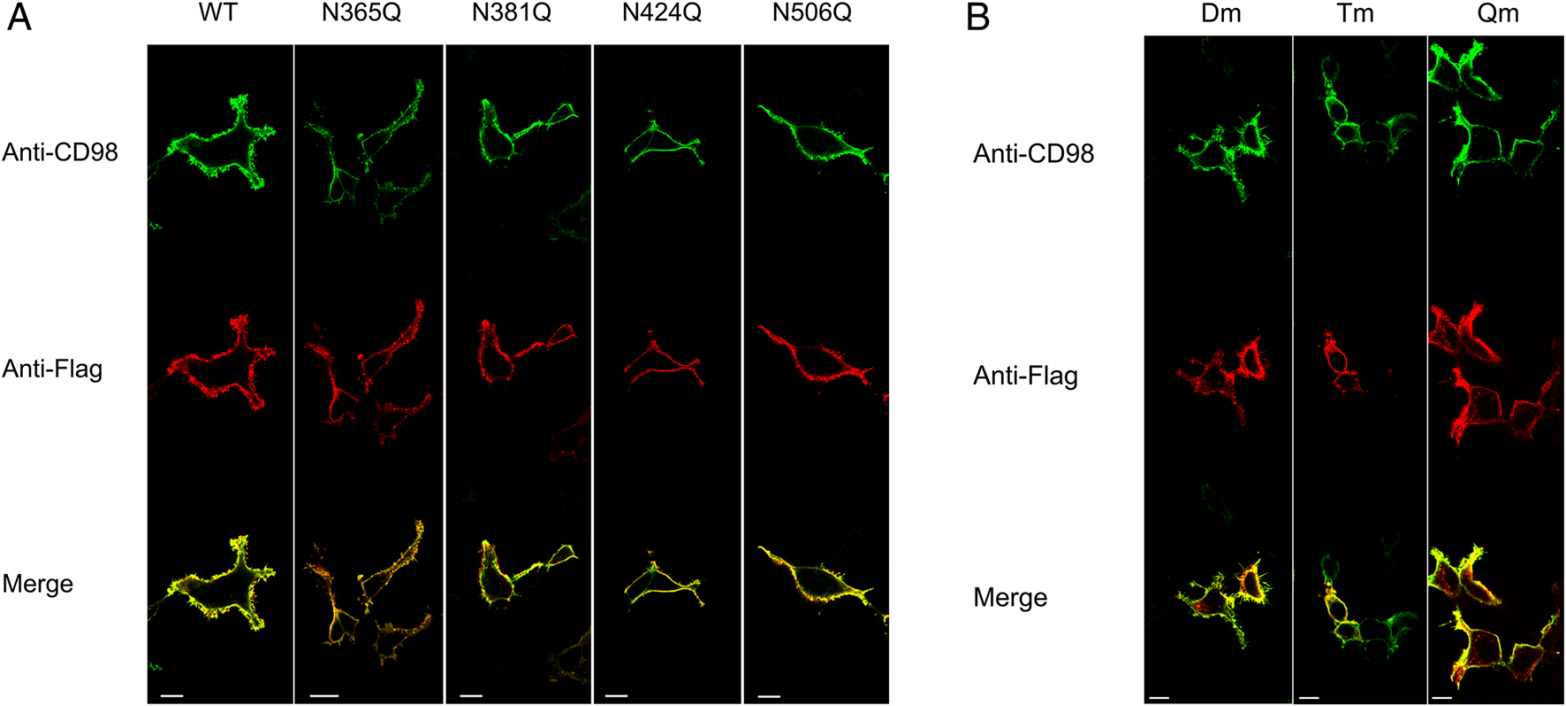
Cell distribution of WT and N-glycosylation mutants of CD98. Cells were transiently transfected with WT or mutant constructs. After 24 h, cells were fixed onto the slide and stained with anti-CD98^18^ that recognizes the epitope protruding towards the extracellular side. After extensive washing the samples were permeabilized using Triton X100 and stained with the anti-FLAG that recognizes the tag located at the N-terminus^33^. Samples were then analyzed by confocal microscopy. Confocal images showed a single, representative, section of a Z-series taken through the entire cell. WT and single mutants were shown in panel (**A**), while double (Dm: N381/424Q), triple (Tm: N365/381/424Q), and quadruple (Qm: N365/381/424/506Q) mutants were shown in panel (**B**). Scale bar 10 µm.

**Figure 4:**
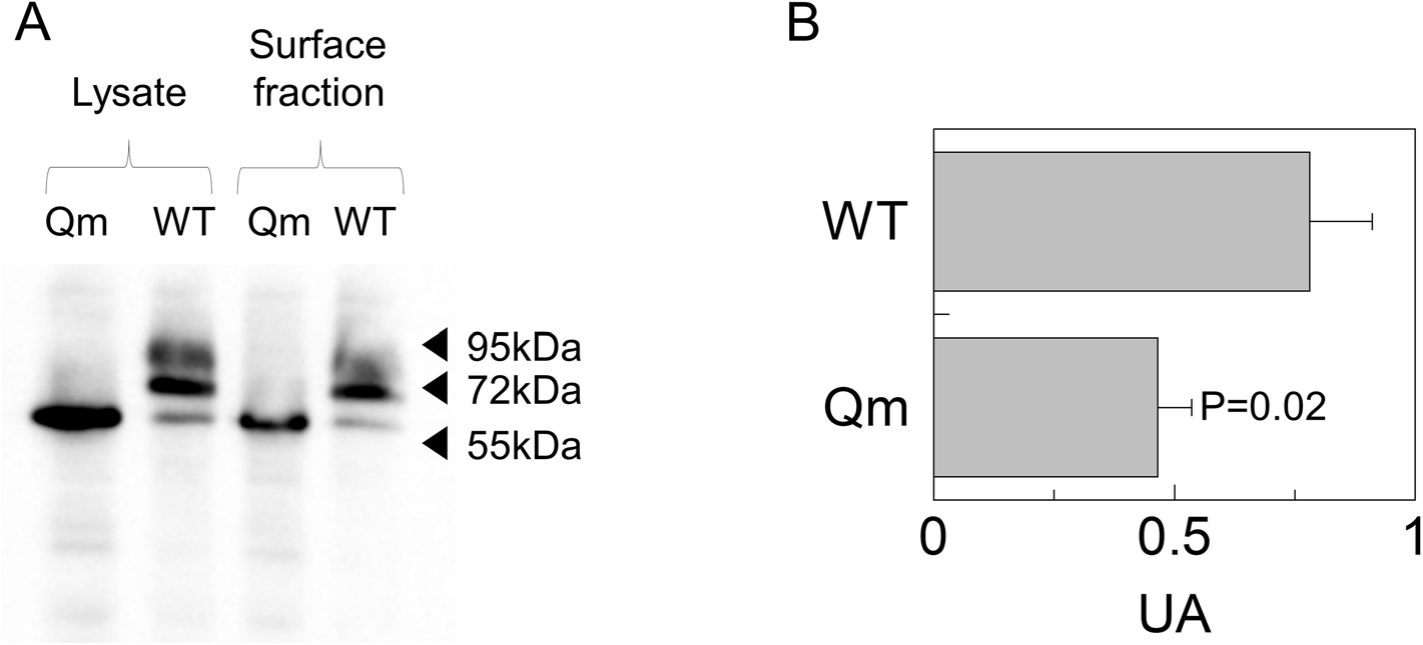
Cell surface biotinylation of CD98. Surface protein fractions isolated as described in material and methods were entirely loaded on SDS-PAGE for western blot analysis; total lysate used to perform membrane protein fraction isolation was shown as a control. Loading order: Qm total lysate (lane1), WT total lysate (lane 2), Qm surface fraction (lane 3) and WT surface fraction (line 4). The western blot was performed using anti-FLAG^33^. The image is representative of three independent experiments. The histogram represents mean of values obtained by scanning densitometry of immunoblots. Statistical analysis was performed for each construct measuring membrane fraction with respect to the total lysate by Student's t-test.

The mutant constructs were transiently transfected into HEK293 cells to assess the expression of hCD98 at the cell membrane by confocal microscopy using two different antibodies for detecting the total over-expressed protein (anti-FLAG) or the membrane localised CD98 (anti-CD98). The detection with anti-CD98 was used as an internal control of the membrane surface since the antibody epitope is extracellular (Fig. 1). As shown in Fig. 3A, each of the single mutants did not show any appreciable difference in membrane localization compared to the WT. Among the multiple mutants (Fig. 3B), the Dm (N381/424Q) and the Tm (N365/381/424Q) exhibited some differences with respect to the WT. The Qm showed the most evident difference in membrane localization compared to the WT (Fig. 3B). In particular, the anti-FLAG images (red) showed several protein spots in intracellular sites that were not present in the anti-CD98 images (green). The merge of the two fluorescence signals highlighted the presence of the non-merged spots in the endocellular area (Fig. 3B). To confirm the data from confocal microscopy, a biotinylation assay was performed. The molecular mass of the Qm correlated well with the lack of glycosyl moieties of this protein (Fig. 4A). A clear decrease in the amount of Qm present in the membrane was observed with respect to the total protein (Fig. 4A, lines 1 and 3). Whereas, in the case of WT only a small difference between total and membrane protein was observed. Indeed, the ability of Qm to reach the membrane is significantly impaired (Fig. 4B). Similar results were obtained treating cells transfected with CD98-WT construct with tunicamycin before brefeldin assay. Indeed, tunicamycin treatment abolished in vivo CD98 N-glycosylation impairing the trafficking of the unglycosylated protein to the plasma membrane (Fig. S1).

**Figure 5:**
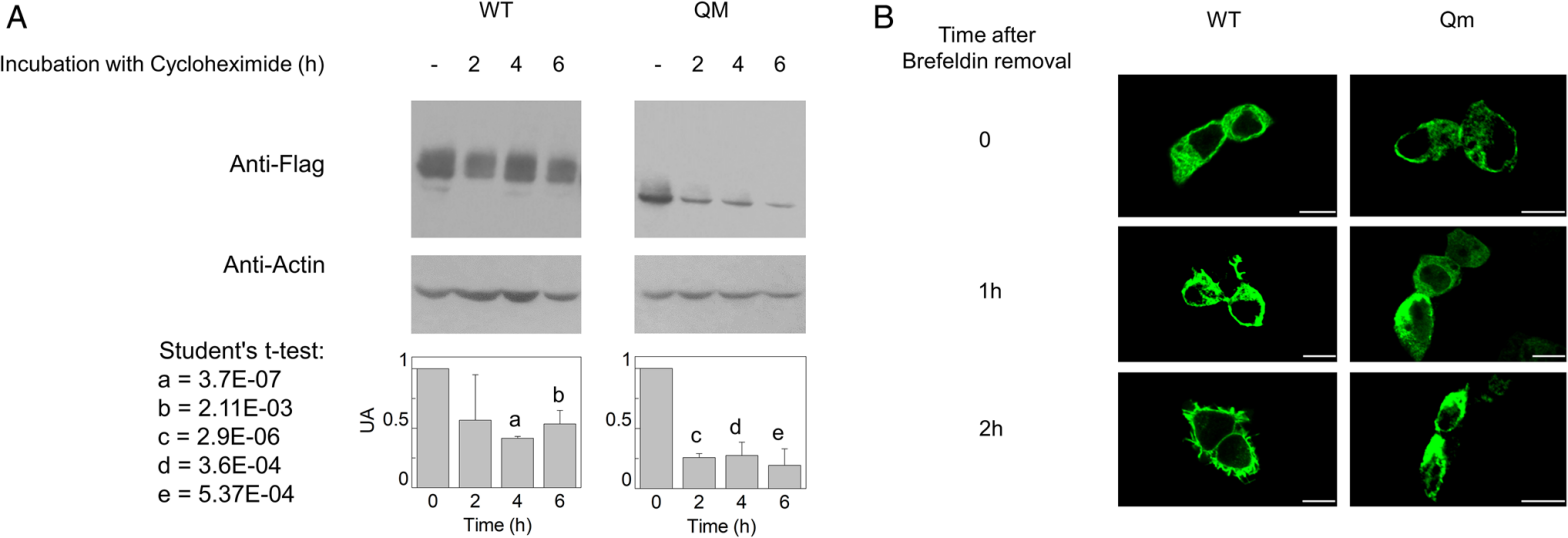
Stability and trafficking from ER to the plasma membrane. In (**A**), transiently transfected HEK293 cells were cultured in the presence of cycloheximide (20 μg/ml) for 0, 2, 4, and 6 h. Then, cells were lysed and analyzed by western blot using anti FLAG-antibody^33^. Anti actin is used as loading control^33^. The western blot image is representative of three independent experiments. The histogram represents mean of values obtained by scanning densitometry of immunoblots; the quantification was performed for WT or Qm comparing each time to the control (time = 0). Data from all experiments are shown in Supplementary Table S1. Significantly differences were estimated by Student's t-test (a = 3.7E−07, b = 0.00211, c = 2.9E−06, d = 0.00036 and e = 0.000537). In (**B**), HEK293 cells transiently transfected with WT or Qm constructs were treated with 5 μM BFA for 7 h. The treatment blocks the protein trafficking toward the plasma membrane allowing for the retention of CD98 into the ER. The rescue of the protein trafficking was obtained by washing out BFA. Cells were fixed and labeled at the indicated times. Confocal images showed a single, representative, section of a Z-series taken through the entire cell. Scale bar 10 µm.

Therefore, to further investigate the trafficking process, only the Qm was used in comparison with the WT. To follow the process of protein migration towards the membrane, HEK293 transiently transfected with WT or Qm construct, were treated with brefeldin that blocks the vesicle trafficking from Golgi towards the plasma membrane and used in confocal analyses^33^. In order to find optimal detection times after brefeldin treatment, the stability of the protein was firstly assessed by treating cells with cycloheximide, a well acknowledged inhibitor of protein synthesis. The amount of WT protein was still higher than 50% after 6 h of protein synthesis block (Fig. 5A and Table S1); whereas, in the case of the Qm a strong decrease was detected already after 2 h and, after 6 h, the protein was less than 20% of the initial amount (Fig. 5A and Table S1). This data demonstrated that N-glycosylation is important for protein stability. Moreover, data from Fig. 5A suggested to not prolong the observation in confocal analysis for more than 2 h after brefeldin treatment. As shown by Fig. 5B and Fig. S2, at time zero, most of the protein was in the cytosol both in the WT and Qm expressing HEK293 cells. After 1 h and, more evidently, 2 h, the WT mostly reached the plasma membrane, whereas the Qm was still localized into the cytosol, clearly demonstrating that in the absence of N-glycosyl residues, the protein capacity to migrate towards the plasma membrane was strongly impaired. The localization of WT and Qm before brefeldin treatment was shown in the upper panel of Fig. S2 as control.

**Figure 6:**
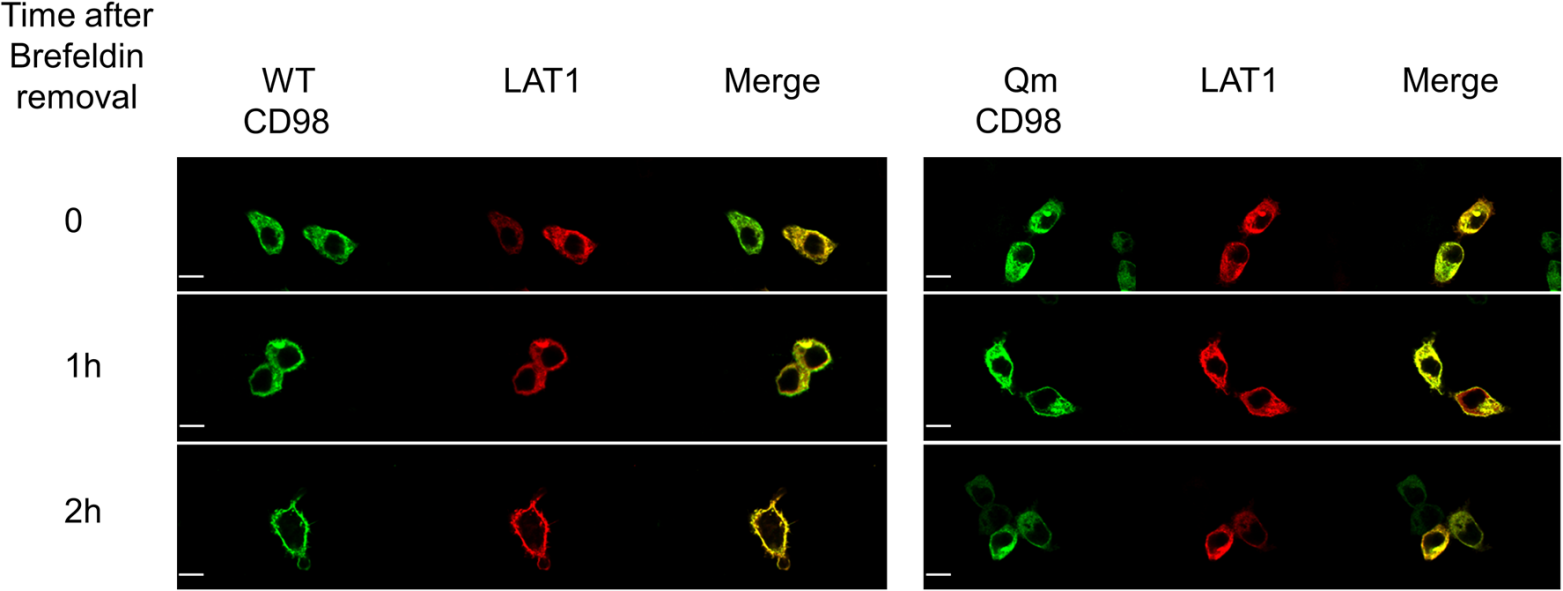
Influence of CD98 trafficking on LAT1 cell distribution. HEK293 cells were transiently co-transfected with LAT1 and WT-CD98 or Qm-CD98 constructs. After 7 h of BFA treatment, cells were washed, fixed and labeled at the indicated times as described in materials and methods. Confocal images show a single, representative, section of a Z-series taken through the entire cell. Scale bar 10 µm.

Given that CD98 can form a functional heterodimer with LAT1, the effect of CD98 N-glycosylation on the trafficking of LAT1 towards the plasma membrane was also investigated. HEK293 cells were transiently co-transfected with LAT1 and alternatively with WT-CD98 or Qm-CD98; LAT1 was mainly localised in at the plasma membrane (Fig. S3A). After co-transfection, the treatment with brefeldin was performed to follow protein migration towards the plasma membrane. As shown by Fig. 6 and Fig. S3, LAT1 followed CD98 routing to the plasma membrane. In the case of co-transfection with WT-CD98, after 2 h from the brefeldin removal, LAT1 reached the plasma membrane. On the contrary, in the case of co-transfection with the Qm-CD98, LAT1 was mainly retained in the cytosol. To exclude any influence of the endogenous CD98 on the LAT1 trafficking, HEK293 cells were transfected with the sole LAT1 construct. In this case, the over-expressed LAT1 lost the capability to reach the plasma membrane (Fig. S4). Indeed, the endogenous expression of CD98 is negligible when compared to the overexpressed one (Fig. S5 left panel). Moreover, the co-transfection of CD98 and LAT1 lead to the expression of both the protein in a good and comparable amount (Fig. S5 right panel).

**Figure 7:**
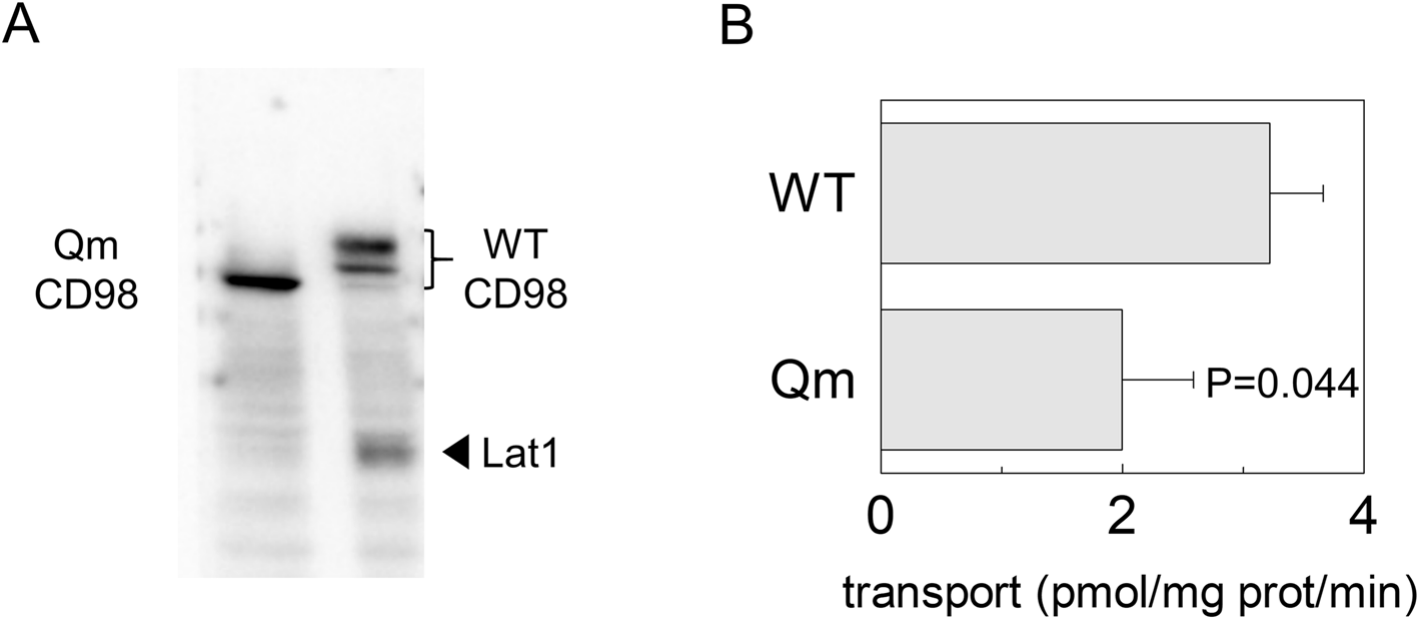
Cell surface biotinylation of CD98 and LAT1. In (**A**), Surface protein fractions isolated from HEK293 transfected with WT-CD98 (lane1) or Qm-CD98 (lane 2) constructs as described in material and methods. The obtained samples were tested for the presence of native LAT1 using an anti-LAT1 antibody^18^. WT and Qm mutant were also tested to ensure that sufficient amount of membrane protein fraction was loaded in the gel using anti-FLAG antibody^33^. The western blot image is representative of three independent experiments. In (**B**), Uptake of [^3^H]-histidine in HEK293 cells. HEK293 cells were seeded and transfected with WT-CD98 or Qm-CD98 constructs as described in materials and methods; transport was started by adding 5 μM [^3^H]-histidine to transfected cells. The transport was blocked after 1 min, by placing cells on ice and washing them with cold transport buffer as described in materials and methods. Results are means ± SD of three independent experiments. Significantly different from WT as estimated by Student's t-test.

These data were confirmed by biotinylation assay; indeed, only the membrane fraction derived from cells transfected with WT-CD98 also contained LAT1 (Fig. 7A).

In agreement with these findings, a transport assay was performed to test LAT1 function using one of the preferred LAT1 substrates, histidine^18^. The uptake of radiolabelled histidine was followed in cells transfected with WT-CD98 or Qm-CD98 (Fig. 7B). In good agreement with analyses performed on biotinylated samples (Figs. 6 and 7A), histidine uptake was significantly lower in cells transfected with Qm-CD98 compared to cells transfected with WT-CD98.

## Discussion

In the present work, the role of N-glycosylation on the trafficking of CD98 towards the plasma membrane was assessed. The strategy for targeting CD98 made use of an anti-CD98 antibody against an epitope that is exclusively located on the extracellular side of the protein and an anti-FLAG antibody which, on the contrary, specifically binds to an intracellular site (Fig. 1). This allowed us to unequivocally visualize the protein which was located at the membrane surface (without permeabilizing cells), deriving from the endogenous protein plus the over-expressed one. On the other hand, the anti-FLAG staining (after cell permeabilization) highlighted the sole over-expressed protein, harbouring the FLAG tag at the N-terminus (Fig. 1). The virtual complete overlapping of the two staining protocols indicated that the endogenous protein does not interfere with the staining of the over-expressed one. Most importantly, the overlap of the two staining signals, confirmed that the over-expressed protein normally reaches the definitive location and that, the FLAG tag does not interfere with the overall trafficking mechanism; indeed, no accumulation of FLAG signal was unspecifically observed in intracellular organelles or the cytosol. This data validated both the over-expression system for the study of CD98 localization and the use of mutants for investigating the role of N-glycosylation, in the absence of interferences from the WT endogenous protein. Moreover, to better appreciate the role of N-glycosyl residues in the dynamics of CD98 trafficking, we employed a well-acknowledged combined approach of brefeldin and cycloheximide^33,34^ that block cell trafficking and de novo protein synthesis, respectively. Therefore, from both WB analysis and confocal microscopy of WT and CD98 site-directed mutants some milestones were reached: (i) N-glycosylation was certainly involved in the protein trafficking to the cell surface (Figs. 3 and 4); (ii) most of the N-glycosylation moieties had to be removed to sensibly reduce the protein residence at the cell surface (Fig. 4), as demonstrated by the little effect of a single mutation on the overall trafficking process (Fig. 3); (iii) N-glycosyl moieties were also involved in the stabilization of the protein at the plasma membrane. Indeed, the lack of N-glycosylated residues triggered a much faster protein degradation, according to its improper localization (Fig. 5A). This finding is in good agreement with the hypothesis that the un-glycosylated protein is more accessible by proteases according to previous findings^35^. Thus, it can be hypothesized that the spots present in HEK293 cells transfected with the CD98-QM construct (Fig. 3B), result from a partial retention into the endoplasmic reticulum, and then followed by proteasomal degradation as previously suggested^30^. Another aspect is that, at least in the analyzed cell context, the major determinant of glycosylation process of CD98 is the N-glycosylation, since the PNGase treatment caused a dramatic shift of the apparent molecular mass of CD98 to that of the sole protein moiety (Fig. 2). We cannot exclude that in other cell contexts, both physiological and pathological, other glycosylation moieties, such as O-glycosylation, may concur to the Post-Translational Modifications (PTMs) of CD98. Bioinformatic predictions obtained through the NetOGlyc—4.0 server, which produces neural network predictions of mucin type GalNAc O-glycosylation sites in mammalian proteins, suggests the presence of O-glycosyl moieties attached to the S504. Furthermore, GlyGen, a database that retrieves information from multiple international data sources, shows the presence of an O-linked glycan residue in CD98; however, this information comes from proteomic studies that didn’t clarify the exact location of the O-Glycosylated residue. It has to be stressed that, O-glycosylation is mainly related to cell processes other than trafficking. This PTM was described in glycocalyx formation, host-microbes interaction, protection from proteolytic cleavage^36^ and in several pathological conditions, including cancer^37^, and cardiovascular disease^38^.

## Conclusion

Taken together our results, we can conclude that the major role of N-glycosylation in routing CD98 to the plasma membrane is here definitively demonstrated. The importance of understanding the molecular basis of CD98 trafficking lies for different reasons: as stated in the introduction, CD98 is a multifunctional protein and its presence/stability in the plasma membrane may affect several cell processes. In pathological conditions, such as cancer, CD98 has a pro-proliferative role due to interaction with integrin and adhesion pathways. Therefore, the possibility of preventing or blocking CD98 residence at the cell surface by acting on its trafficking, may result in slowing down uncontrolled cell growth/metastasis^39^. Moreover, a role in cell metabolism is also expected considering the formation of a functional heterodimer with some of the SLC7 members. These transporters are unglycosylated proteins and the chaperone role of CD98 in routing them to the plasma membranes was already proposed^19,27,40^. In this scenario, our data are in perfect agreement with the previous observations; more importantly, we here clarify the molecular basis of the chaperone function played by CD98. By combining confocal analysis, biotinylation and transport assays we demonstrated that one of the most studied SLC7 members, i.e., LAT1, follows CD98 to reach the plasma membrane. We may hypothesize that a similar scenario may occur in the case of the other SLC7 members known to interact with CD98, given that the heterodimer is formed at the level of two conserved residues^17,21^. This finding opens also other perspectives when considering that a therapy acting on CD98 trafficking will surely impair the LAT1 chaperoning, with consequent high relevance to human therapy. Then, CD98 inhibition would act synergistically with specific LAT1 inhibitors, such as the tyrosine derivative JPH203 that is in human trial for anticancer therapy^41^.

## Materials and methods

### Cloning and mutagenesis

The cDNA of hCD98 was subcloned into pSF-CMV-NEO-NH2-3XFLAG, a 5913 bp vector that established transient expression of N-terminal FLAG fusion protein in the mammalian cell. The vector encodes a three-FLAG epitope at the N-terminus and contains a CMV promoter. The WT hCD98 construct was used to introduce mutations using the Phusion Site-Directed Mutagenesis Kit by Thermo Scientific. All mutations were verified by DNA sequencing. The HA-LAT1 DNA sequence was subcloned into the pCDNA3.1 mammalian expression vector.

### Cell culture

HEK293 cells (ATCC) were grown on 75 cm^2^ dish using Dulbecco's Modified Eagle Medium (DMEM) supplemented with 10% (v/v) fetal bovine serum (FBS), 1 mM glutamine, 1 mM sodium pyruvate, and pen/strep as antibiotics. Cells were maintained at 37 °C in a humidified incubator, 5% CO_2_ atmosphere.

### SDS–polyacrylamide gel electrophoresis and western blotting

HEK 293 cells were solubilized in RIPA buffer (20 mM Tris–HCl pH 7.5, 150 mM NaCl, 1 mM EDTA, 1 mM EGTA, 1% TX-100, 1% sodium deoxycholate) supplemented with protease inhibitors. The proteins were separated on a 12% polyacrylamide gel and transferred to a nitrocellulose membrane. After blocking, the membrane was incubated with a mouse anti-FLAG (1:1000) or a rabbit anti-LAT1 (1:1000) overnight at 4 °C, respectively. The blot was washed three times at room temperature and incubated with the appropriate secondary antibody (1:10,000) for 1 h and revealed by chemiluminescence.

### In vitro endoglycosidase treatment

Cells expressing WT and mutants were solubilized as described above. Fifty μg of total lysate were treated with a denaturing buffer and incubated for 5 min at 90 °C. After cooling, N-glycosidase F from Merck was added to the lysate and left for 3 h at 37 °C. Aliquots were used for SDS-PAGE, followed by immunoblotting analysis.

### Evaluation of protein stability

HEK293 cells were seeded on 6-well plates and cultured to 80% confluence. Then, cells were transfected with 1 µg WT and Qm constructs using PolyJet according to the manufacturer's procedures. Cells were incubated with cycloheximide (20 μg/ml) to inhibit protein synthesis. At increasing time points, cells were harvested, lysed, and subjected to SDS-PAGE followed by immunoblot analysis.

### Confocal microscopy

HEK293 cells were seeded onto the coverslip and cultured until 60% of confluence. According to the manufacturer's procedures, cells were transfected with 1 µg hLAT1, hCD98 WT and mutant constructs using PolyJet transfection reagent. After 24 h, cells onto the coverslip were fixed with 4% (w/v) paraformaldehyde (15 min; 37 °C), rinsed three times with PBS, and incubated with blocking buffer (10% bovine serum albumin in PBS) for 1 h at room temperature. Then, cells were incubated with the rabbit monoclonal anti-CD98 (1:250) as internal proof that the protein is located at the plasma membrane. The recognized epitope protrudes towards the extracellular milieu; hence, the antibody can also work without membrane permeabilization. After treatment with the first antibody, samples were extensively washed. Membrane was permeabilized with 0.1% Triton-X100, and incubation for 1 h with mouse monoclonal anti-FLAG antibody (1:500), whose epitope is located inside the cell (Fig. 1), was performed. The LAT1 labelling was performed with a post-permeabilization incubation of cells with mouse monoclonal anti-HA (1:500). Alexa Fluor 594-conjugated anti-mouse or Alexa Fluor 488-conjugated anti-rabbit secondary antibodies were used with 1:300 dilution. The coverslips were then washed and mounted with Moviol mounting medium. The cellular trafficking and localization of labelled epitopes were visualized by Confocal Laser Scanning Microscopy (CLSM, Fluoview FV300, Olympus Italia, Segrate (MI), Italy) in the z-scan mode (step size: 0.25 µm). To evaluate ER to Golgi trafficking, transfected cells were incubated with 5 μM Brefeldin-A for 7 h to inhibit ER to Golgi protein trafficking. Then, cells were rinsed with PBS to washout BFA, removing the inhibition. Finally, cells were placed in FBS-free DMEM containing cycloheximide (1 mM). Times of cycloheximide incubation were chosen based on the protein stability tests described before.

### Tunicamycin treatment

HEK293 cells were seeded on poly-D-lysine-coated 6-well plates and cultured up to 80% confluence. Then, cells were transfected with 1 µg hCD98 WT using PolyJet transfection reagent. After 6 h of transfection, tunicamycin (1 µg/mL) or vehicle (DMSO), was added to the complete medium and cells were grown up to 24 h. After tunicamycin incubation, cells were subjected to biotinylation procedure, as described below.

### Biotinylation

HEK293 cells were seeded on poly-D-lysine-coated 6-well plates and cultured up to 80% confluence. Cells were transfected with 1 µg hCD98 WT and Qm constructs using PolyJet according to the manufacturer's procedures. After 24 h incubation, cells were washed twice in ice-cold PBS and incubated with 1 ml of 0.5 mg/ml biotin in PBS for 45 min at 4 °C. Then, cells were incubated for 30 min with PBS/Glycine buffer to quench all non-reacting biotin. After ice-cold PBS washing, cells were solubilized with RIPA buffer and incubated overnight with streptavidin agarose beads. Beads were then washed and centrifugated to isolate proteins were that located in the plasma membrane.

### Other methods

The homology structural model of hCD98 was built through Modeller 10.2, using the CryoEM structure of CD98^21^, lacking the N-terminus, and the N-terminal ab initio model of CD98 from Rosetta. The glycosylation sites were predicted by NetNGlyc and NetOGlyc Server. The amount of protein in the total lysate was measured with the Lowry-Folin assay. The quantification of protein bands detected by immunoblotting was estimated by using the Chemidoc imaging system.

### Transport measurements in cells

HEK 293 cells were seeded onto 12 well plates coated with Poly-D-Lysine and cultured until they reached 60% confluence. Then, cells were transfected with Polyjet transfection reagent according to the manufacturer's procedures with 0.5 µg of WT-CD98 and Qm constructs. After 24 h transfection, cells were used for transport assay of [^3^H]-histidine. In brief, cells were washed twice with a warm transport buffer, composed of 20 mM Tris HCl pH 7.5. Transport was started by adding radiolabelled 5 µM [^3^H]-histidine and terminated after one minute by washing out the transport buffer and rinsing the cells three times with the same ice-cold transport buffer (0.5 ml per well per rinse). Then, cells were solubilized in 500 µL of 1% TX-100 solution, and 400 µL cell extracts were counted for radioactivity. The remaining 100 µL in each well was used for measuring total protein concentration. LAT1-histidine transport was evaluated by subtracting the transport values of each condition from those deriving from blank, i.e., samples treated with the well-known LAT1 specific inhibitor BCH. In particular, 10 mM BCH was added at time zero together with radiolabelled [^3^H]-histidine in the transport buffer.

## Supplementary Information

Supplementary Information 1.

Supplementary Information 2.

## Data Availability

The datasets analysed during the current study are available from the corresponding author on reasonable request.

## Abbreviations

LAT1: Large neutral amino acids transporter small subunit 1
LAT2: Large neutral amino acids transporter small subunit 2
xCT: Cystine/glutamate transporter
PNGase: N-Glycosidase F
HEK293: Human embryonic kidney 293 cells
DMEM: Dulbecco's modified eagle medium
Tris: Tris(hydroxymethyl)aminomethane
FBS: Fetal bovine serum
EDTA: Ethylenediaminetetraacetic acid
TX-100: T-Octylphenoxypolyethoxyethanol
PBS: Phosphate buffered saline
BCH: 2-Amino-2-norbornanecarboxylic acid

## References

[1] Deves R, Boyd CA (2000). Surface antigen CD98(4F2): Not a single membrane protein, but a family of proteins with multiple functions. J. Membr. Biol..

[2] Haynes BF, Hemler ME, Mann DL, Eisenbarth GS, Shelhamer J, Mostowski HS (1981). Characterization of a monoclonal antibody (4F2) that binds to human monocytes and to a subset of activated lymphocytes. J. Immunol..

[3] Yagita H, Hashimoto Y (1986). Monoclonal antibodies that inhibit activation and proliferation of lymphocytes II: Requisite role of the monoclonal antibody-defined antigen systems in activation and proliferation of human and rat lymphocytes. J. Immunol..

[4] Gottesdiener KM, Karpinski BA, Lindsten T, Strominger JL, Jones NH, Thompson CB (1988). Isolation and structural characterization of the human 4F2 heavy-chain gene, an inducible gene involved in T-lymphocyte activation. Mol. Cell Biol..

[5] Porter JC, Hogg N (1998). Integrins take partners: Cross-talk between integrins and other membrane receptors. Trends Cell Biol..

[6] Feral CC, Nishiya N, Fenczik CA, Stuhlmann H, Slepak M, Ginsberg MH (2005). CD98hc (SLC3A2) mediates integrin signaling. Proc. Natl. Acad. Sci. USA..

[7] Estrach S, Lee SA, Boulter E, Pisano S, Errante A, Tissot FS (2014). CD98hc (SLC3A2) loss protects against ras-driven tumorigenesis by modulating integrin-mediated mechanotransduction. Cancer Res..

[8] Boulter E, Estrach S, Tissot FS, Hennrich ML, Tosello L, Cailleteau L (2018). Cell metabolism regulates integrin mechanosensing via an SLC3A2-dependent sphingolipid biosynthesis pathway. Nat. Commun..

[9] Zent R, Fenczik CA, Calderwood DA, Liu S, Dellos M, Ginsberg MH (2000). Class- and splice variant-specific association of CD98 with integrin beta cytoplasmic domains. J. Biol. Chem..

[10] Almutairi SM, Ali AK, He W, Yang DS, Ghorbani P, Wang L (2019). Interleukin-18 up-regulates amino acid transporters and facilitates amino acid-induced mTORC1 activation in natural killer cells. J. Biol. Chem..

[11] Hirohata Y, Arii J, Liu Z, Shindo K, Oyama M, Kozuka-Hata H (2015). Herpes simplex virus 1 recruits CD98 heavy chain and beta1 integrin to the nuclear membrane for viral de-envelopment. J. Virol..

[12] Malleret B, El Sahili A, Tay MZ, Carissimo G, Ong ASM, Novera W (2021). Plasmodium vivax binds host CD98hc (SLC3A2) to enter immature red blood cells. Nat. Microbiol..

[13] Wells RG, Lee WS, Kanai Y, Leiden JM, Hediger MA (1992). The 4F2 antigen heavy chain induces uptake of neutral and dibasic amino acids in Xenopus oocytes. J. Biol. Chem..

[14] Cano-Crespo S, Chillaron J, Junza A, Fernandez-Miranda G, Garcia J, Polte C (2019). CD98hc (SLC3A2) sustains amino acid and nucleotide availability for cell cycle progression. Sci. Rep..

[15] Digomann D, Linge A, Dubrovska A (2019). SLC3A2/CD98hc, autophagy and tumor radioresistance: a link confirmed. Autophagy.

[16] Verrey F, Jack DL, Paulsen IT, Saier MH, Pfeiffer R (1999). New glycoprotein-associated amino acid transporters. J Membr Biol..

[17] Palacin M, Nunes V, Font-Llitjos M, Jimenez-Vidal M, Fort J, Gasol E (2005). The genetics of heteromeric amino acid transporters. Physiology (Bethesda)..

[18] Napolitano L, Scalise M, Galluccio M, Pochini L, Albanese LM, Indiveri C (2015). LAT1 is the transport competent unit of the LAT1/CD98 heterodimeric amino acid transporter. Int. J. Biochem. Cell Biol..

[19] Rosell A, Meury M, Alvarez-Marimon E, Costa M, Perez-Cano L, Zorzano A (2014). Structural bases for the interaction and stabilization of the human amino acid transporter LAT2 with its ancillary protein 4F2hc. Proc. Natl. Acad. Sci. USA..

[20] Lee Y, Wiriyasermkul P, Jin C, Quan L, Ohgaki R, Okuda S (2019). Cryo-EM structure of the human L-type amino acid transporter 1 in complex with glycoprotein CD98hc. Nat. Struct. Mol. Biol..

[21] Yan R, Zhao X, Lei J, Zhou Q (2019). Structure of the human LAT1-4F2hc heteromeric amino acid transporter complex. Nature.

[22] Parker JL, Deme JC, Kolokouris D, Kuteyi G, Biggin PC, Lea SM (2021). Molecular basis for redox control by the human cystine/glutamate antiporter system xc(). Nat. Commun..

[23] Kantipudi S, Jeckelmann JM, Ucurum Z, Bosshart PD, Fotiadis D (2020). The heavy chain 4F2hc modulates the substrate affinity and specificity of the light chains LAT1 and LAT2. Int. J. Mol. Sci..

[24] Scalise M, Console L, Rovella F, Galluccio M, Pochini L, Indiveri C (2020). Membrane transporters for amino acids as players of cancer metabolic rewiring. Cells.

[25] Liu J, Xia X, Huang P (2020). xCT: A critical molecule that links cancer metabolism to redox signaling. Mol. Ther..

[26] Kanai Y (2022). Amino acid transporter LAT1 (SLC7A5) as a molecular target for cancer diagnosis and therapeutics. Pharmacol Ther..

[27] de la Ballina LR, Cano-Crespo S, Gonzalez-Munoz E, Bial S, Estrach S, Cailleteau L (2016). Amino acid transport associated to cluster of differentiation 98 heavy chain (CD98hc) is at the cross-road of oxidative stress and amino acid availability. J. Biol. Chem..

[28] Ip H, Sethi T (2016). CD98 signals controlling tumorigenesis. Int. J. Biochem. Cell Biol..

[29] Papetti M, Herman IM (2001). Controlling tumor-derived and vascular endothelial cell growth: Role of the 4Ff2 cell surface antigen. Am. J. Pathol..

[30] D'Agostino S, Lanzillotta D, Varano M, Botta C, Baldrini A, Bilotta A (2018). The receptor protein tyrosine phosphatase PTPRJ negatively modulates the CD98hc oncoprotein in lung cancer cells. Oncotarget.

[31] Fort J, de la Ballina LR, Burghardt HE, Ferrer-Costa C, Turnay J, Ferrer-Orta C (2007). The structure of human 4F2hc ectodomain provides a model for homodimerization and electrostatic interaction with plasma membrane. J. Biol. Chem..

[32] Gupta R, Brunak S (2002). Prediction of glycosylation across the human proteome and the correlation to protein function. Pac Symp. Biocomput..

[33] Console L, Scalise M, Tarmakova Z, Coe IR, Indiveri C (2015). N-linked glycosylation of human SLC1A5 (ASCT2) transporter is critical for trafficking to membrane. Biochim. Biophys. Acta..

[34] Nivillac NM, Bacani J, Coe IR (2011). The life cycle of human equilibrative nucleoside transporter 1: From ER export to degradation. Exp. Cell. Res..

[35] Esmail S, Manolson MF (2021). Advances in understanding N-glycosylation structure, function, and regulation in health and disease. Eur. J. Cell Biol..

[36] Wandall HH, Nielsen MAI, King-Smith S, de Haan N, Bagdonaite I (2021). Global functions of O-glycosylation: Promises and challenges in O-glycobiology. FEBS J..

[37] Ferrer CM, Sodi VL, Reginato MJ (2016). O-GlcNAcylation in cancer biology: Linking metabolism and signaling. J. Mol. Biol..

[38] Loaeza-Reyes KJ, Zenteno E, Moreno-Rodriguez A, Torres-Rosas R, Argueta-Figueroa L, Salinas-Marin R (2021). An overview of glycosylation and its impact on cardiovascular health and disease. Front. Mol. Biosci..

[39] Maimaiti M, Sakamoto S, Sugiura M, Kanesaka M, Fujimoto A, Matsusaka K (2021). The heavy chain of 4F2 antigen promote prostate cancer progression via SKP-2. Sci Rep..

[40] Cormerais Y, Giuliano S, LeFloch R, Front B, Durivault J, Tambutte E (2016). Genetic disruption of the multifunctional CD98/LAT1 complex demonstrates the key role of essential amino acid transport in the control of mTORC1 and tumor growth. Cancer Res..

[41] Okano N, Hana K, Naruge D, Kawai K, Kobayashi T, Nagashima F (2020). Biomarker analyses in patients with advanced solid tumors treated with the LAT1 inhibitor JPH203. In Vivo.

