# Supplementary material for: N-glycosylation is crucial for trafficking and stability of SLC3A2 (CD98)
Source: Sci Rep. 2022 Aug 26;12:14570. doi: 10.1038/s41598-022-18779-4 (PMC9418156; doi:10.1038/s41598-022-18779-4)
Supplement: Supplementary file 1 — Supplementary Information 1. [file 41598_2022_18779_MOESM1_ESM.pdf]

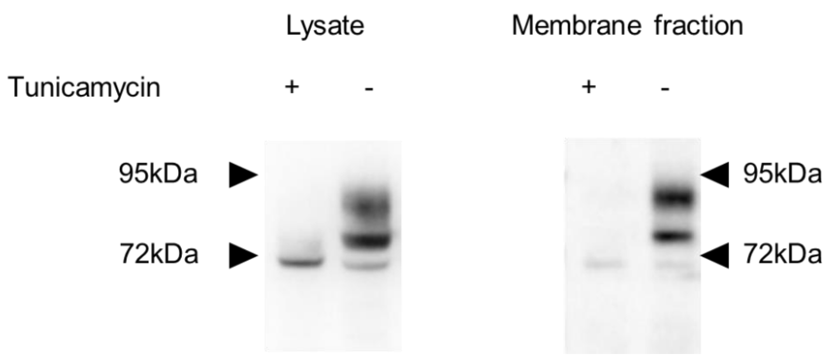

Fig.S1 Tunicamycin treatment and biotinylation assay

Surface protein fractions isolated as described in material and methods were entirely loaded on SDS-PAGE for western blot analysis; total lysates from HEK293 treated with tunicamycin or vehicle, used to perform membrane protein fraction isolation were shown as a control. The western blot was performed using anti-FLAG to reveal the overexpressed CD98. The image is representative of three independent experiments.

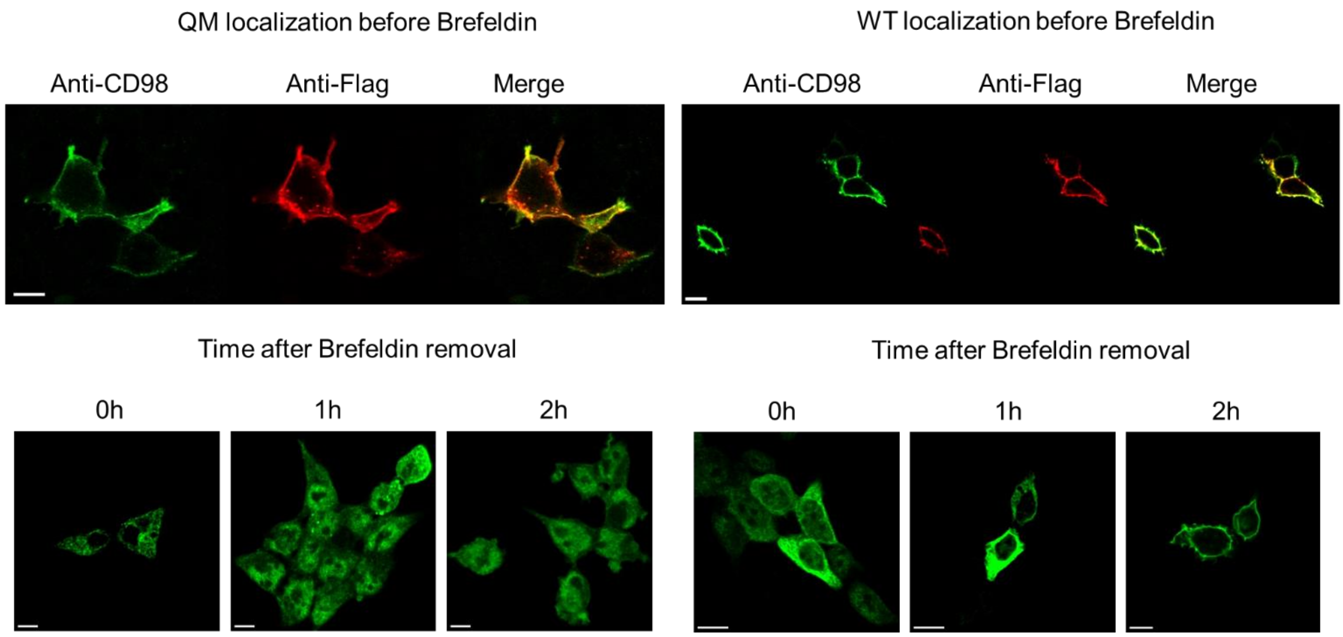

Fig. S2. CD98 traffiking

HEK293 cells were transiently transfected with Qm or WT constructs. The upper panel shows the CD98 localization before cell treatement with 5 μM BFA. The treatment blocks the protein trafficking toward the plasma membrane allowing for the retention of CD98 into the ER (time 0h after brefeldin removal). The rescue of the protein trafficking was obtained by washing out BFA. Cells were fixed and labeled at the indicated times. Confocal images showed a single, representative, section of a Z-series taken through the entire cell. Scale bar: 10μm.

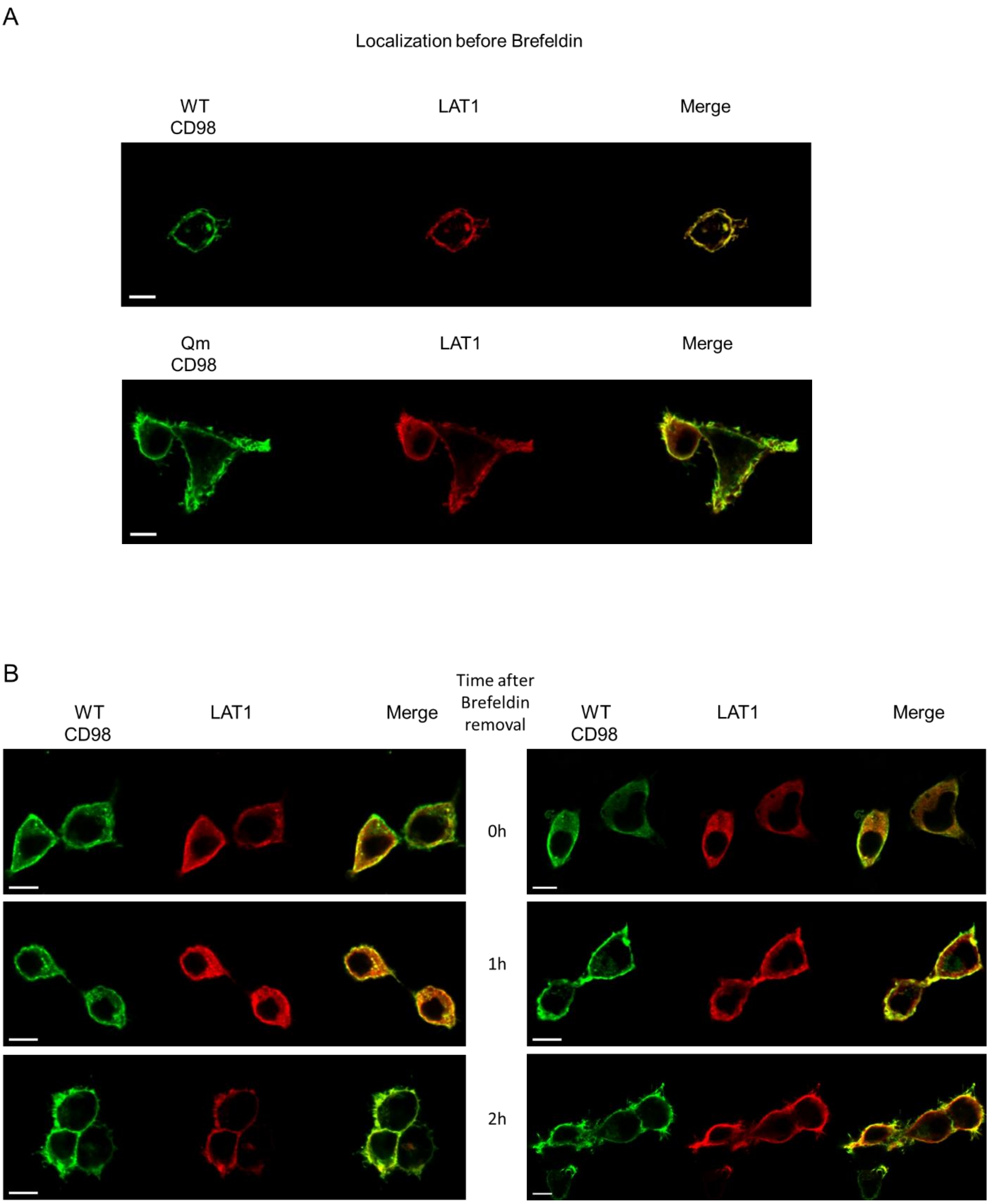
A
Localization before Brefeldin
WT CD98
LAT1
Merge
Qm CD98
LAT1
Merge
B
WT CD98
LAT1
Merge
Time after Brefeldin removal
0h
1h
2h
WT CD98
LAT1
Merge

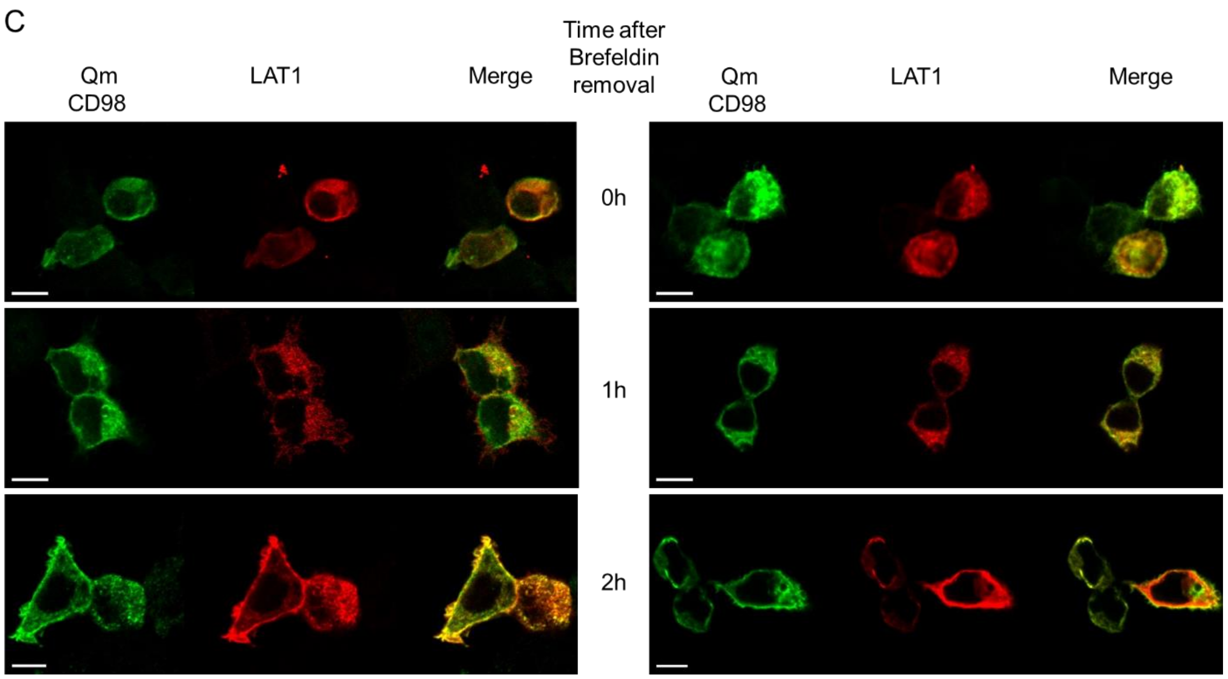

Fig. S3. Influence of CD98 trafficking on LAT1 cell distribution.

HEK293 cells were transiently co-transfected with LAT1 and WT-CD98 or Qm-CD98 constructs. Panel A showed the localization of CD98 and LAT1 before Brefeldin treatment. After 7 h of BFA treatment, cells were washed, fixed and labeled at the indicated times as described in materials and methods. Panel B showed the LAT1/WT-CD98 trafficking; Panel c showed the LAT1/Qm-CD98 trafficking Confocal images show a single, representative, section of a Z-series taken through the entire cell. Scale bar: 10µm.

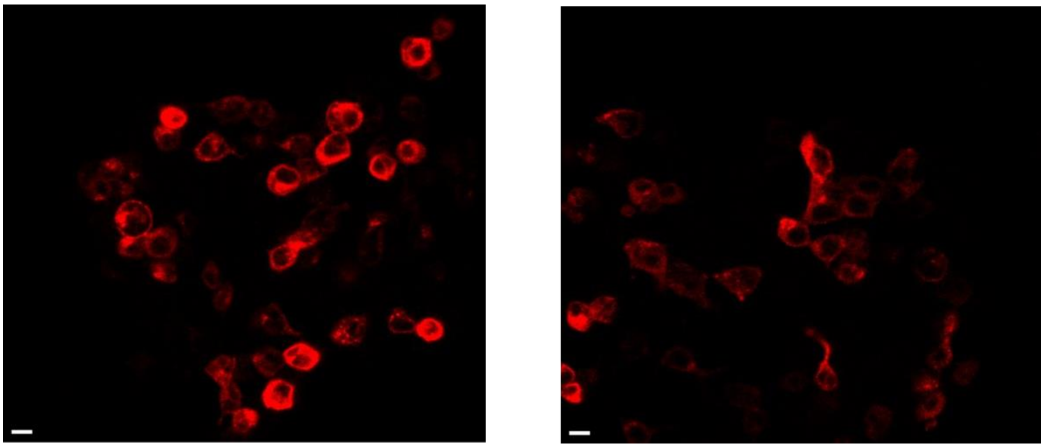

Fig. S4. Cell distribution of the overexpressed LAT1 in absence of the CD98 overexpression.

Cells were transiently transfected with LAT1 construct. After 24 h, cells were fixed onto the slide and stained with anti-HA antibody. Samples were then analyzed by confocal microscopy. Confocal images showed a single, representative, section of a Z-series taken through the entire cell. Scale bar: 10µm.

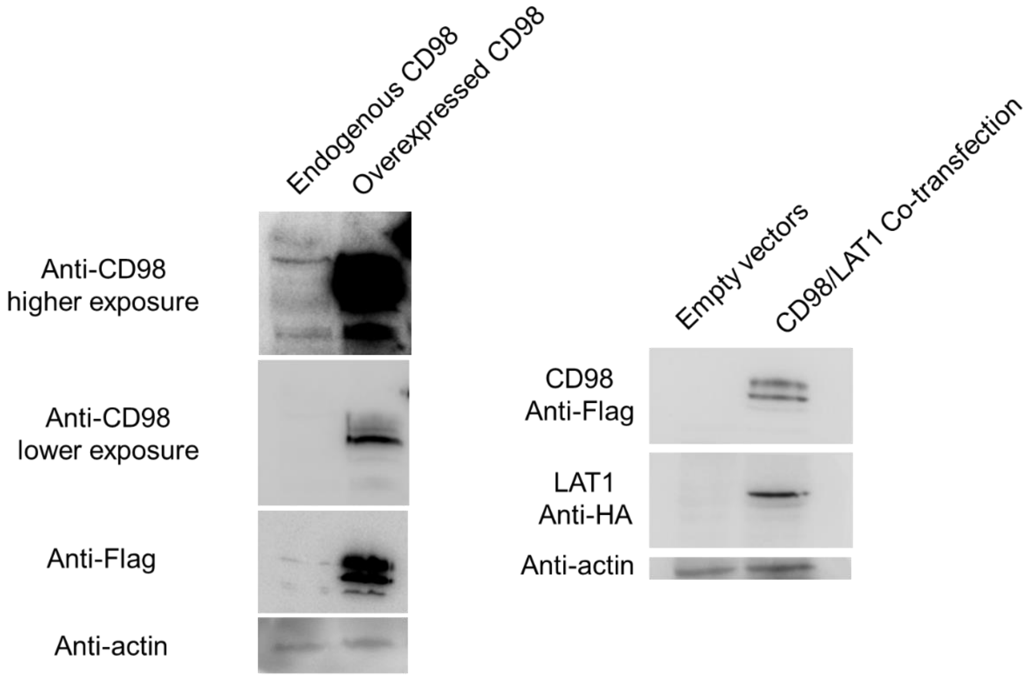

Fig. S5. Western blot analysis of HEK293 cells.

Cell lysates (25 µg) from HEK293 cells, transfected with empty vector or with hCD98 WT or hLAT1 constructs, were loaded on SDS-PAGE for western blot analysis. The immunoblot was performed using: anti-FLAG and anti-HA to reveal the overexpressed CD98 and LAT1 respectively, and anti-CD98 to reveal the endogenous protein. Anti-actin was detected as loading control. The image is representative of three independent experiments.

Table S1. Values obtained by scanning densitometry of three immunoblots including that showed in fig. 5A, performed to test the influence of N-glycosylation on CD98 stability. Data were plotted in fig. 5A.

	1° exp.		2° exp.		3° exp.		1° exp.	2° exp.	3° exp.		
	CD98-Flag	Actin	CD98-Flag	Actin	CD98-Flag	Actin	CD98-Flag /Actin	CD98-Flag /Actin	C CD98-Flag /Actin	Average	S.D.
WT											
0h	1	1	1	1	1	1	1	1	1	1	0
2h	0.555523	1.508309	0.446555	1.366233	1.083669	1.075775	0.368308	0.326852	1.007338	0.567499	0.381475
4h	0.813054	2.000573	0.510892	1.180596	0.695897	1.723307	0.40641	0.43274	0.403815	0.414322	0.016003
6h	0.584761	1.22235	0.575481	0.863083	0.851167	1.841561	0.478391	0.666774	0.462198	0.535788	0.113726
Qm											
0h	1	1	1	1	1	1	1	1	1	1	0
2h	0.27563	0.95905	0.219893	0.828104	0.228308	1.033875	0.287399	0.265537	0.220828	0.257921	0.033933
4h	0.210205	1.14783	0.241346	0.60348	0.187802	0.781504	0.183133	0.399923	0.240309	0.274455	0.112357
6h	0.079355	0.827191	0.163985	0.468495	0.07733	0.585705	0.095933	0.350025	0.132029	0.192663	0.13747
