# Supplementary material for: N-glycosylation is crucial for trafficking and stability of SLC3A2 (CD98)
Source: Sci Rep. 2022 Aug 26;12:14570. doi: 10.1038/s41598-022-18779-4 (PMC9418156; doi:10.1038/s41598-022-18779-4)

Uncropped fig 2

Without PGNaseF

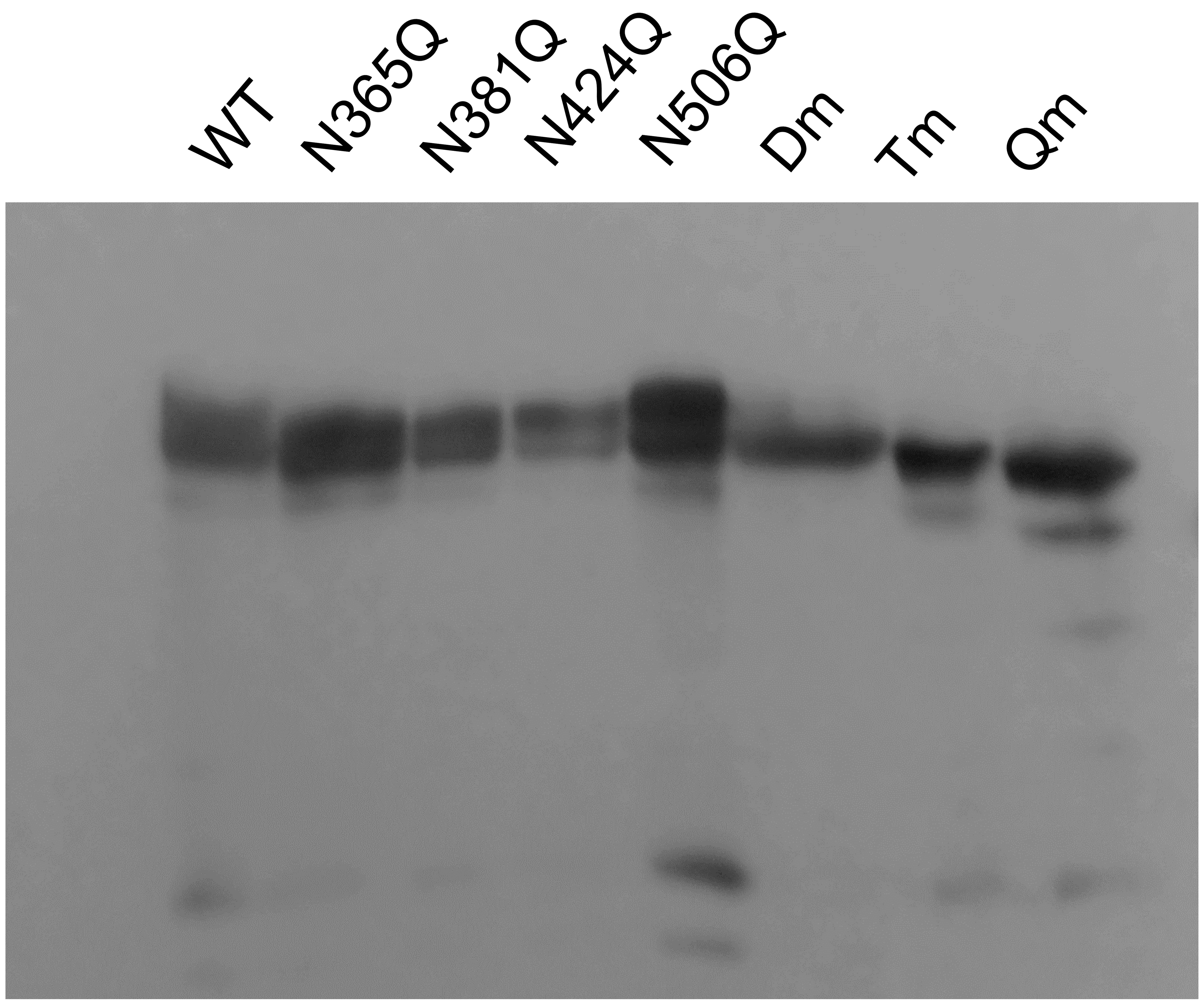

With PGNaseF

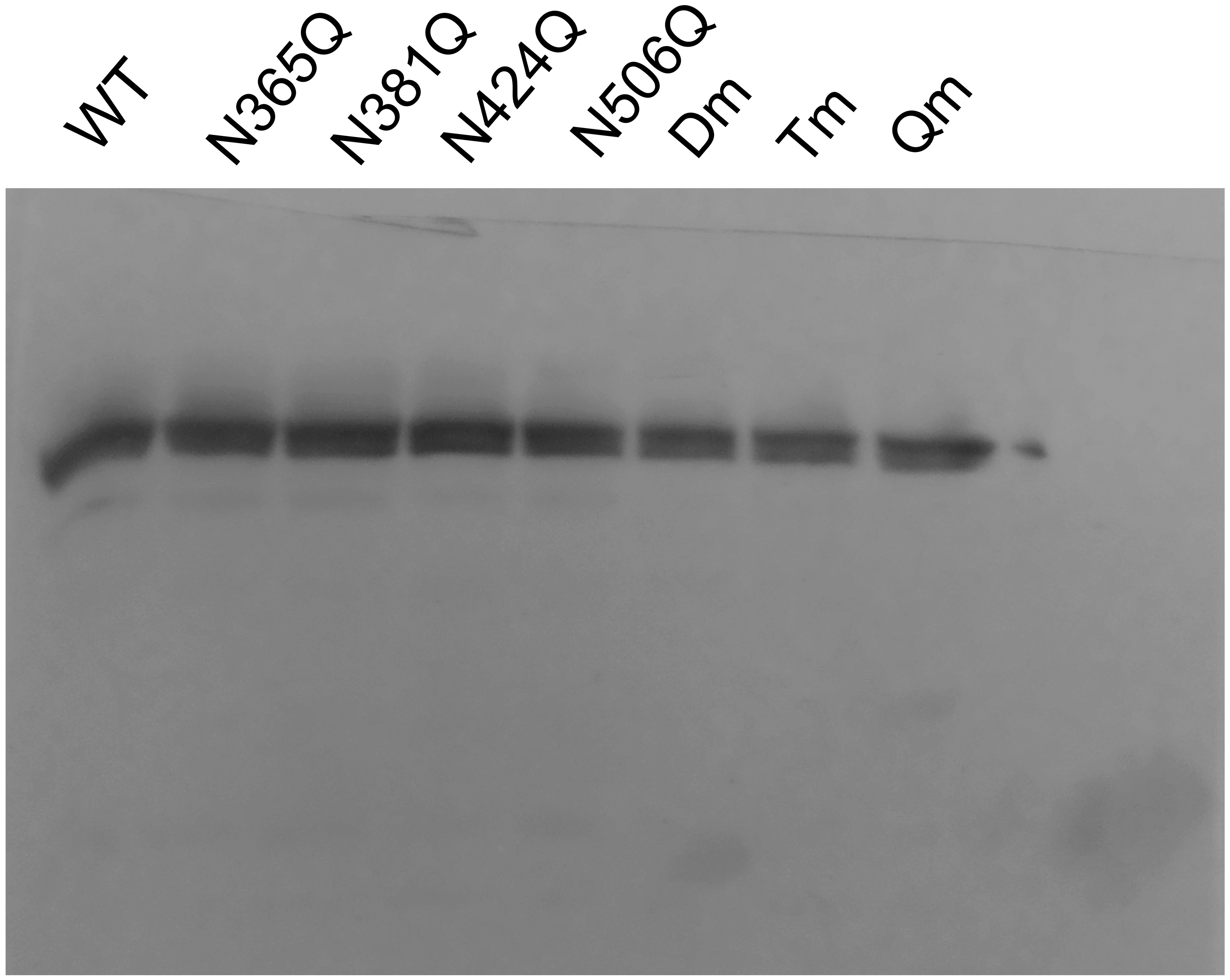

Uncropped fig 4

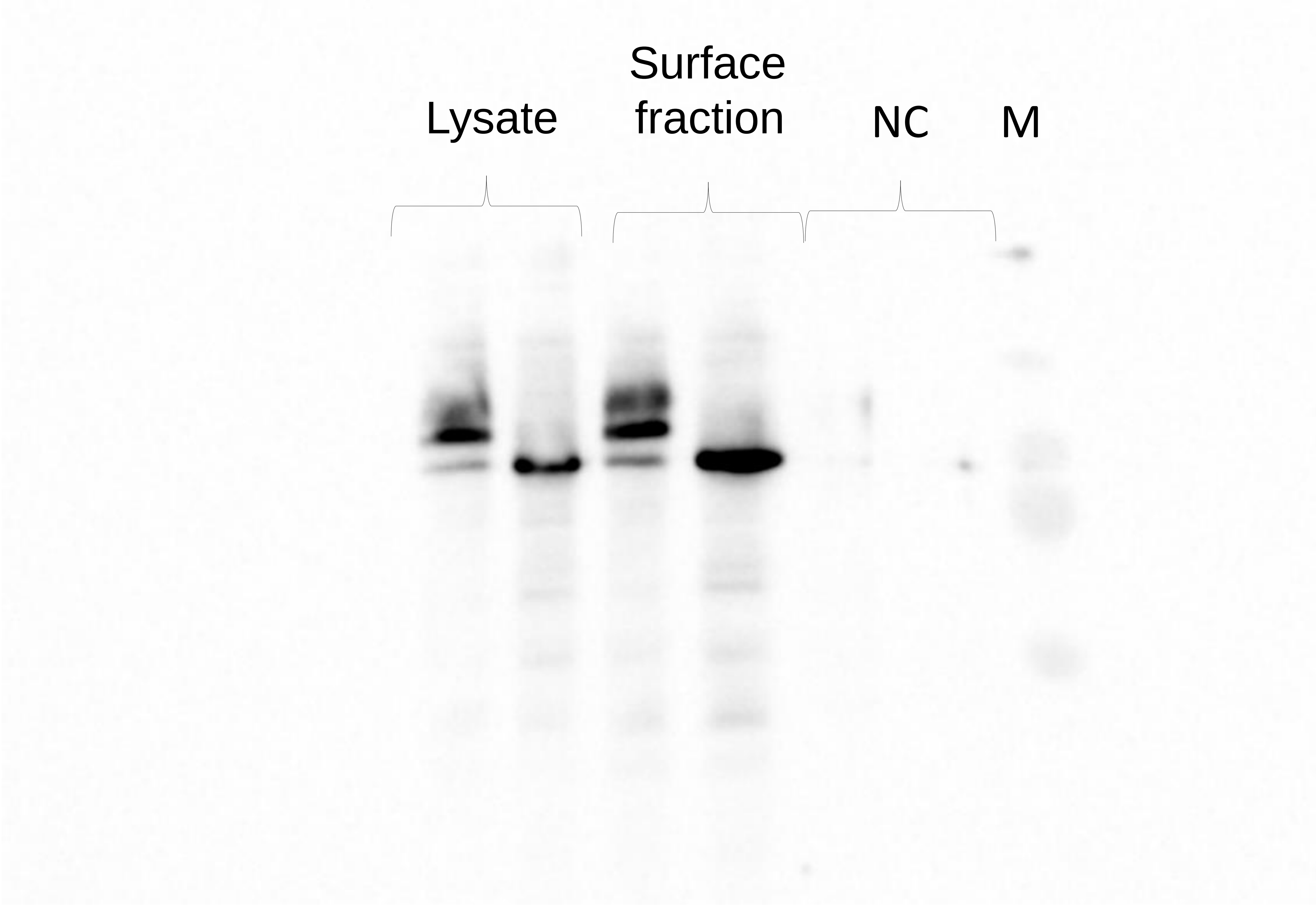

Negative control (NC), Marker (M)

Uncropped fig 5

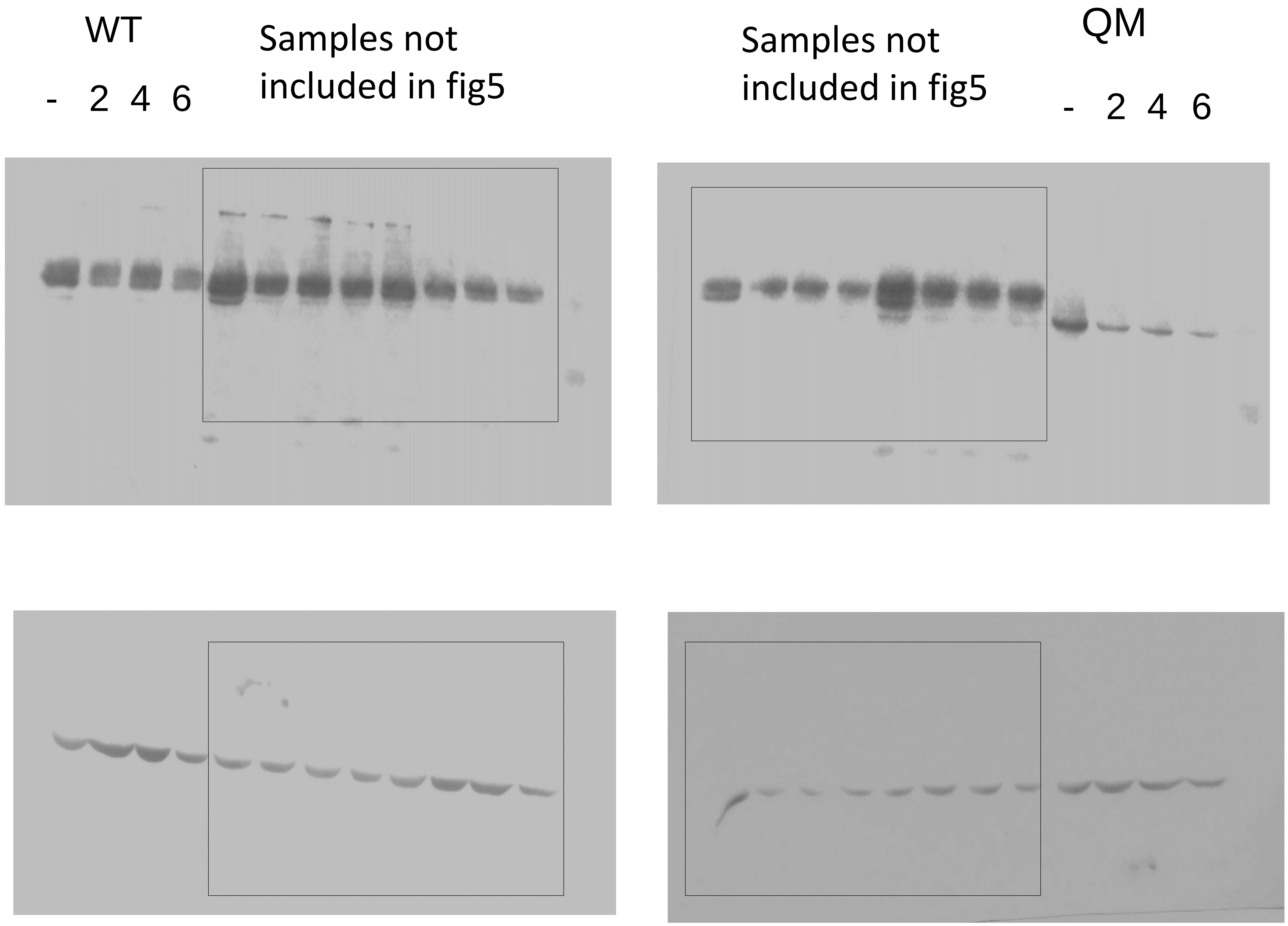

Uncropped fig 7

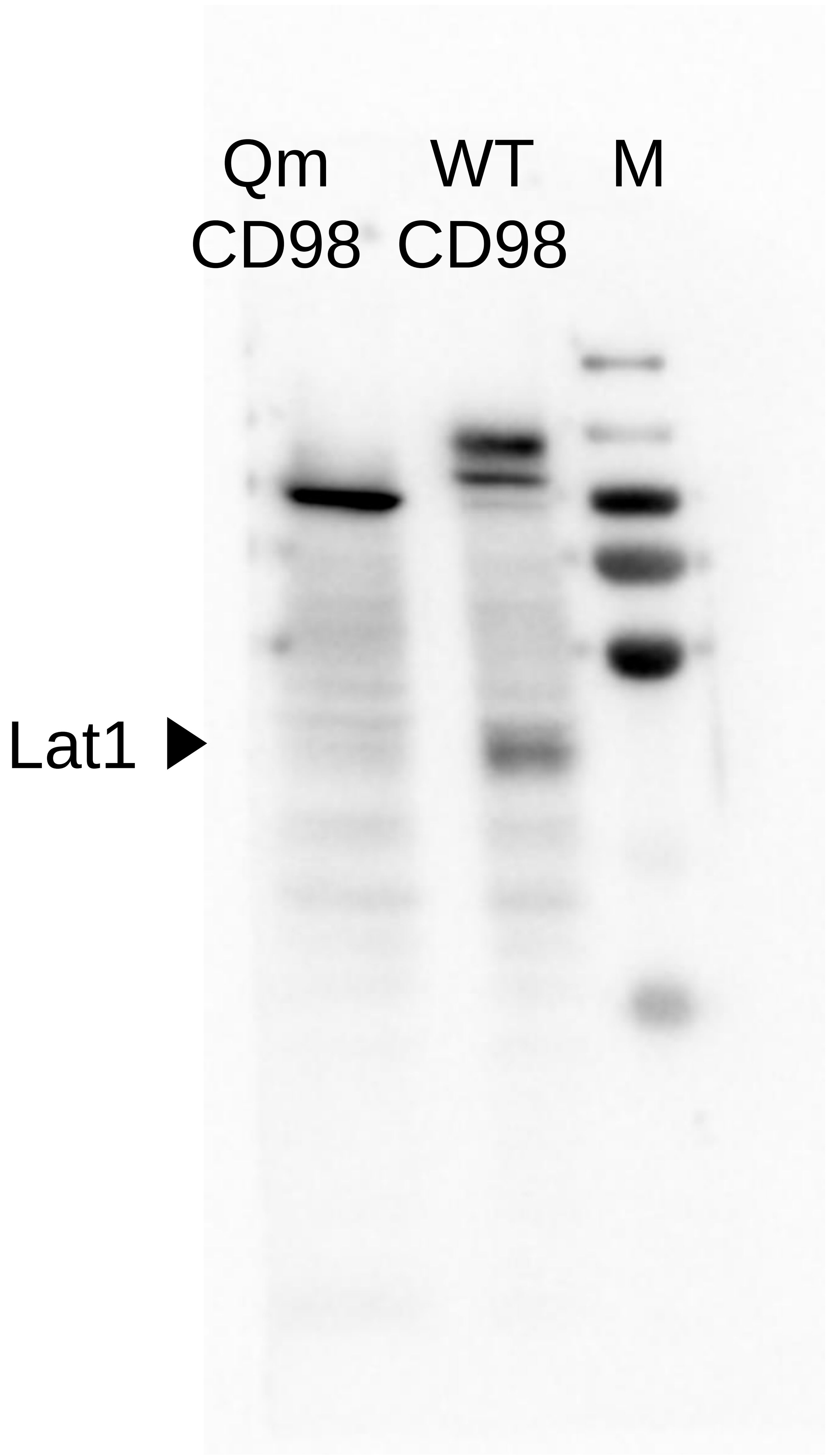

Supplement: Supplementary file 2 — Supplementary Information 2. [file 41598_2022_18779_MOESM2_ESM.pdf]
